# Propofol Infusions and Their Role for Patients Undergoing Surgery for Head and Neck Squamous Cell Carcinoma

**DOI:** 10.7759/cureus.53447

**Published:** 2024-02-02

**Authors:** Matthew Owrey, Kevin J Min, Marc Torjman

**Affiliations:** 1 Anesthesiology, Thomas Jefferson University, Philadelphia, USA

**Keywords:** cancer anesthesia, head and neck cancer surgery, human papillomavirus (hpv), head and neck squamous cell carcinoma (hnscc), sevoflurane vs propofol

## Abstract

Purpose: Propofol infusions may improve survival for patients undergoing surgery for various types of cancer. However, propofol has not been shown to improve survival for all cancer types. The purpose of this retrospective study was to investigate whether propofol infusions during surgery for head and neck squamous cell carcinoma (HNSCC) improved survival.

Methods: A retrospective analysis was performed on all patients undergoing surgery for HNSCC with neck dissection at one institution between June 15, 2017, and April 28, 2021. The primary analysis was performed as a cohort study, with one cohort receiving a propofol infusion and the other cohort not receiving a propofol infusion. A second analysis was performed as a case-control study with matching by cancer staging, human papillomavirus (HPV)/p16 status, pathology margin status, surgical duration within 90 minutes, American Society of Anesthesiologists (ASA) status, and Charlson Comorbidity Index (CCI) within a score of 1. Cases included patients who received a propofol infusion, and controls were patients who did not receive a propofol infusion.

Results: For the primary analysis, there was no statistically significant difference in age (p=0.650), BMI (p=0.956), sex (p=0.069), and CCI (p=0.351), but there was a statistically significant difference in ASA status (p=0.003). The time exposed to sevoflurane (MAC >0.3) was significantly higher in the no-propofol group (p<0.001). The duration of surgery was significantly longer in the propofol patient group compared to the no-propofol group (p=0.013). The length of hospital stay was roughly two days longer for the propofol group (p=0.029). There was no difference in survival for patients who did not receive propofol versus those who did (p=0.247), even after adjusting for HPV/p16 tumor marker status (p=0.223). When patients were matched in a case-control approach, there were no differences in age (p=0.956), BMI (p=0.828), CCI (p=1.000), or ASA status (p=1.000). The death rate was not significant between the cases and controls (p=0.311).

Conclusions: This data suggests that propofol may not influence survival in patients with HNSCC. Larger studies are necessary to better characterize the effect of propofol infusions on patients with HNSCC.

## Introduction

Propofol is among the most commonly used anesthetics to provide surgical anesthesia and has multiple properties that add to its utility, including vasodilation and anti-emetic effects [1,2]. Evidence has emerged that suggests propofol also possesses potent anti-tumor properties and works to combat tumor progression through both direct and indirect mechanisms [3,4]. For example, propofol modulates complex epigenetic pathways related to miRNA, IncRNA, and histone acetylation as well as genetic signaling pathways like HIF-2𝛼, NF-𝜅B, MAPK, SLUG, and Nrf2 to suppress various mechanisms of cancer growth and evolution [3-6]. Studies have also demonstrated its ability to enhance the activity of natural killer cells and helper T-cells, reduce proinflammatory cytokines, and inhibit prostaglandin production [7,8]. Altogether, there is evidence suggesting that propofol may have utility beyond maintaining or inducing anesthesia.

The effects seen at the cellular and biochemical levels have been supported by a number of retrospective studies evaluating the relationships between propofol, cancer recurrence, and survival. However, these findings have been inconsistent. Studies have suggested that colon, gastric, esophageal, glioblastoma, hepatocellular, intrahepatic cholangiocarcinoma, pancreatic, and prostate cancer may be more likely to benefit from total intravenous anesthesia (TIVA) with propofol [9-17]. In studies of patients undergoing surgery for high-grade glioma, non-small cell lung cancers, and breast cancer, maintenance anesthesia with propofol did not appear to have any impact on survival when compared to volatile anesthetics [18-21]. The variability in response to propofol suggests that the biochemical profile of the cancer plays a major role in propofol’s anticancer efficacy. It is critical to determine which types of cancer respond well to propofol or poorly to volatile anesthetics in order to better tailor the perioperative management of cancer patients.

One cancer type that has not been extensively evaluated regarding its response to propofol is head and neck squamous cell carcinoma (HNSCC). HNSCC, which is highly associated with human papillomavirus (HPV), carries a dismal prognosis despite surgical intervention [22]. More than 60% of those diagnosed with HNSCC present with stage III or IV disease, which is characterized by invasion into adjacent structures and local metastases, and of those who achieve disease remission, upwards of 50% of patients have recurrence within two years of their primary treatment [23,24]. Many patients with HNSCC undergo surgical resection of their primary tumor with an accompanying neck dissection to remove lymph nodes. In general, patients with clinically positive lymph node involvement or an advanced tumor stage, such as stage III or IV, undergo neck dissection, with the exception of those whose tumors are deemed unresectable [25]. These are often long procedures, typically several hours in duration. Therefore, patients may be exposed to high doses of propofol or an inhaled anesthetic. Until recently, there have been few studies evaluating whether propofol improves survival in patients undergoing surgery for HNSCC. Therefore, the purpose of this study was to evaluate whether patients undergoing radical neck dissections for HNSCC had an improvement in overall survival when receiving maintenance anesthesia with propofol infusions.

## Materials and methods

This is a retrospective study of all head and neck cancer patients who had a neck dissection performed at Thomas Jefferson University Hospital between June 15, 2017, and April 28, 2021. The study was approved by the Jefferson Institutional Review Board (control number: 21E.034), and a waiver of consent was obtained. This article is in accordance with the STROBE standards of reporting for observational studies.

Participants and data sources

**Figure 1:**
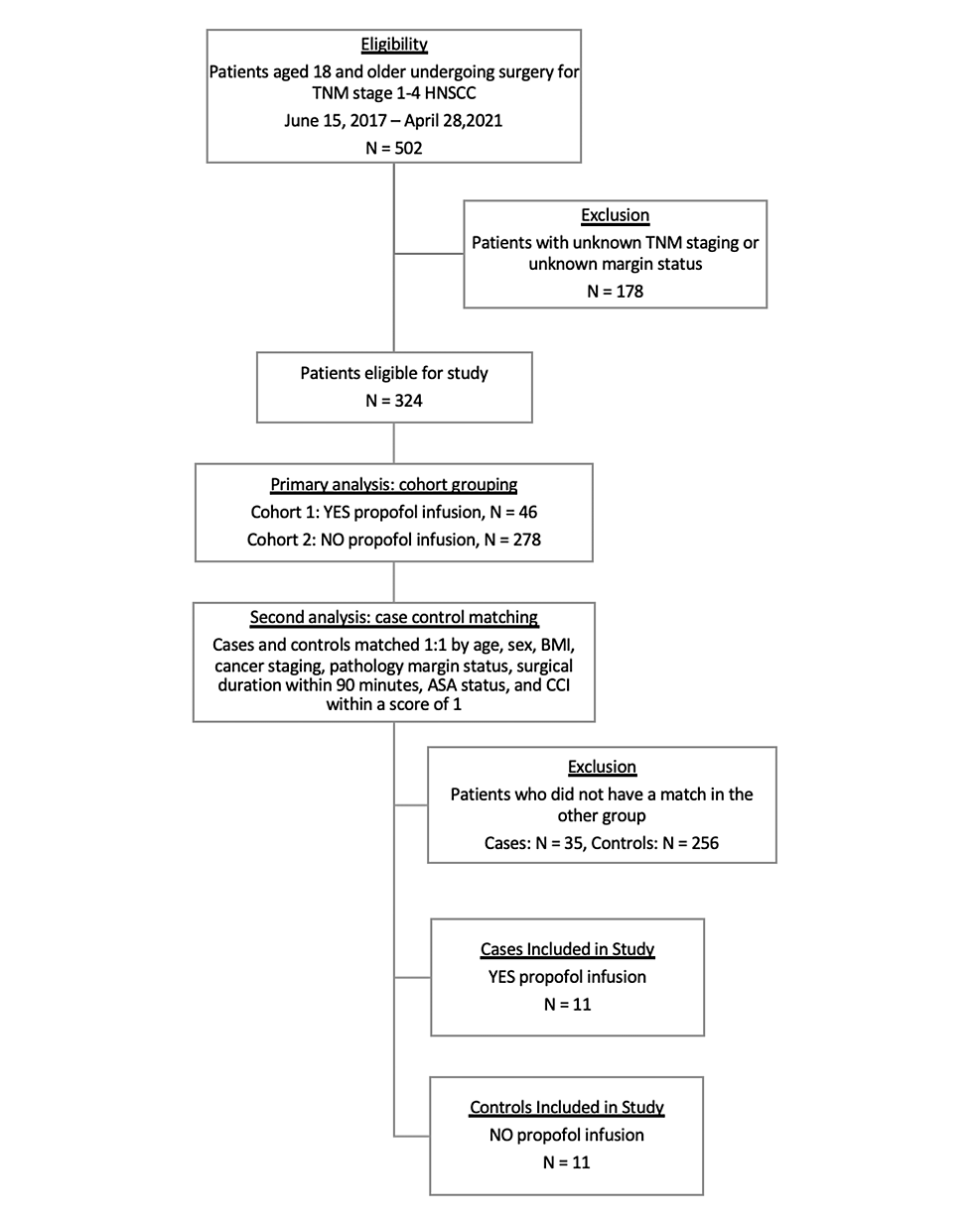
Flow diagram for patient selection Flow diagram illustrating the patient selection process and criteria for patient exclusion for the cohort analysis and the case-control analysis.

Patient information was collected via institutional EPIC electronic medical records (EMR). The date of death was gathered from the National Death Index (NDI; https://www.cdc.gov/nchs/ndi/index.htm). Patients above the age of 18 who underwent elective head and neck surgery for TNM stage 1-4 squamous cell carcinoma (SCC) between the dates of June 15, 2017, and April 28, 2021, were included in the study. Patients were excluded for insufficient data regarding TNM staging and margin status. The patient selection and criteria for patient exclusion for the primary analysis and the second analysis are illustrated in the flow diagram (Figure 1).

Variables

The following patient information was collected: sex, age at the time of surgery, BMI, ASA physical status, duration of surgery, dexamethasone dose, staging of the primary tumor, metabolic equivalents greater than or less than 4, positive margins, use of adjuvant chemotherapy, postoperative recurrence, days of death since admission for surgery, hospital length of stay (HLOS), duration with propofol infusion rate >25 mcg/kg/min, total propofol dosage, and duration of exhaled concentration of sevoflurane >0.3. Patient comorbidities were quantified using the Charlson Comorbidity Index (CCI).

Matching and outcome measurements

Of the 502 patients in the original sample, 324 patients were included in the study. For the primary analysis, patients were divided into two cohorts. Cohort 1 consisted of 46 patients who were induced with 1-2.5 mg/kg propofol and received maintenance anesthesia with propofol infusions alone or propofol infusions with inhaled sevoflurane. Cohort 2 consisted of 278 patients who were induced with 1-2.5 mg/kg propofol and received only inhaled sevoflurane for maintenance. A second analysis was conducted as a case-control series after reviewing patients’ medical records. Cases included patients receiving propofol infusions, while controls included patients who did not receive propofol infusions. Cases and controls were carefully matched 1:1 based on age, sex, BMI, ASA, CCI within a score of 1, and surgical duration within 90 minutes. Patients were also matched based on cancer staging, HPVp16 status, and pathology margins. Primary outcomes include mortality rate and median survival days from surgery between propofol infusion and no propofol infusion groups. Secondary outcomes include survival based on HPV/p16 status.

Statistical analysis

Data are presented as means ± SD and medians (IQR) as indicated. Data were analyzed using the Student t-test, the Mann-Whitney U test, the Fisher exact test, binary logistic regression analysis, and Kaplan-Meier survival analysis. Two-sided tests were used, with p<0.05 set for statistical significance. SYSTAT version 13 (Grafiti LLC, Palo Alto, CA, USA) and SPSS Statistics version 29 (IBM Corp. Released 2022. IBM SPSS Statistics for Windows, Version 29.0. Armonk, NY: IBM Corp.) software were used to perform the statistical analyses.

## Results

The original study sample consisted of 502 patients who had head and neck cancer surgery with neck dissection between the dates of June 15, 2017, and April 28, 2021. There were 178 patients excluded from the study analysis due to a lack of TNM staging data or unknown margin status. Of the 324 patients included in the study, 46 received a propofol infusion, while 278 did not receive a propofol infusion. These patients were further stratified into cases and controls for a case-control analysis (Figure 1).

Primary analysis

When comparing no propofol infusion vs. propofol infusion groups, there was no significant difference in age (mean 65.45±12.21 vs. 64.44±14.17, p=0.650) and BMI (mean 28.25±6.47 vs. 28.31±5.55, p=0.956). ASA status was significantly (p=0.003) different among the two groups, explained by approximately 10% less ASA 2 and 8% more ASA 4 patients in the propofol infusion group. There was no significant (p=0.069) difference in sex distribution between the two groups. There was also no significant difference in the CCI when comparing no propofol infusion vs. propofol infusion groups (mean 4.84±1.77 vs. 5.15±2.06, p=0.351). The duration of surgery was significantly longer in the propofol infusion group compared to the no propofol infusion group (mean 474.50±228.15 vs. 382.65±197.25 min, p=0.013). The HLOS was approximately two days longer for the propofol infusion group (median (IQR) 2.0 (4) and 4.0 (5.25), p=0.029). The time exposed to sevoflurane (MAC >0.3) was significantly higher in the no propofol infusion group (median (IQR) 337.5 (303.75) and 75.0 (393.75), p<0.001).

**Figure 2:**
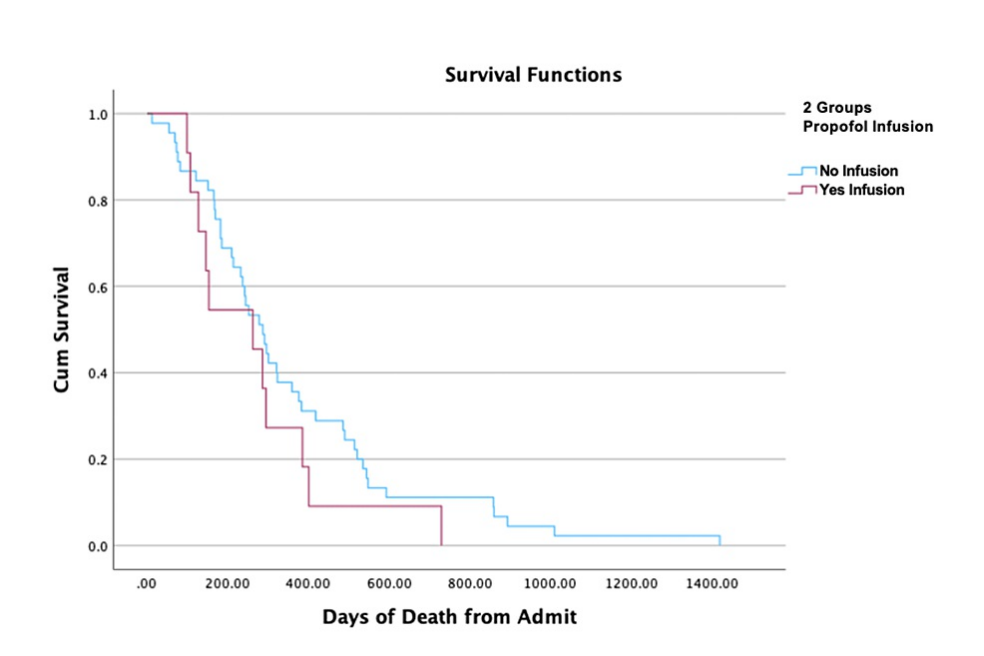
Effect of propofol infusions on survival Kaplan-Meier survival curve for patients receiving propofol infusions during surgery (red line) compared to those not receiving propofol infusions (blue line). There was no statistically significant difference in survival between groups (p=0.247).

The overall death rate was not significant between the no propofol infusion and propofol infusion groups (16.7% vs. 23.9%, p=0.295). The number of survival days from the day of surgery until death was not significantly different between the no propofol infusion and propofol infusion groups (median (IQR); 287.00 (332.00) vs. 262.00 (258.00), log-rank Mantel-Cox p=0.247). The Kaplan-Meier survival curves for the two groups are shown in Figure 2.

**Figure 3:**
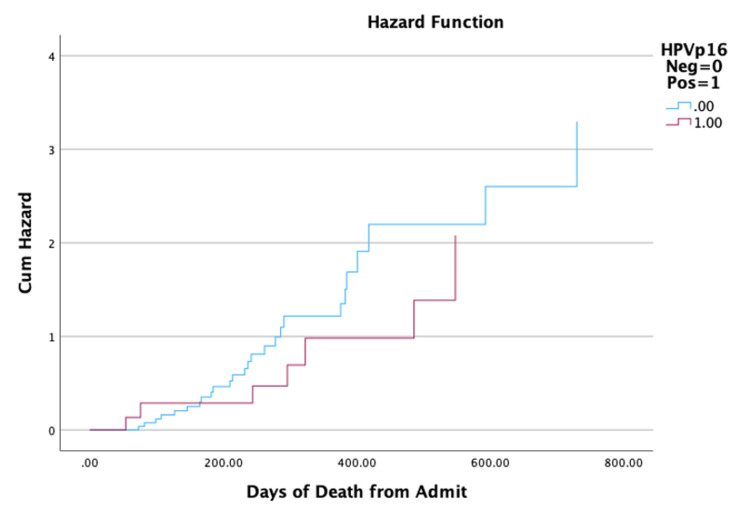
Effect of HPV status on hazard rate Hazard function depicting survival days from surgery depending on HPV/p16 status. There was no statistically significant difference in survival days from surgery based on HPV/p16 status (p=0.242). HPV: human papillomavirus

There was no significant (p=0.295) difference in survival days from the time of surgery until death between HPV/p16-positive and HPV/p16-negative patients (median (IQR); 323.00 (506.00) vs. 234.50 (215.75), log-rank Mantel-Cox p=0.242), as shown in Figure 3. The mortality rate was significantly lower in HPV/p16-positive patients (8% vs. 27%, p<0.001); however, mortality was not significantly (p=0.223) affected by propofol infusion after controlling for HPV/p16 status.

Case-control analysis

To further explore our findings, we performed a second analysis using a case-control approach with careful matching of subjects on the following key variables: age, sex, BMI, ASA, CCI within a score of 1, and surgical duration within 90 minutes. Subjects were also matched based on cancer staging, HPV/p16 data, and pathology margin status.

Out of the initial 324 patients, we obtained 11 cases receiving a propofol infusion and 11 controls not receiving a propofol infusion (N=22) for this analysis. There were no significant differences in mean age between cases and controls (64.75±8.86 vs. 64.54±9.00, p=0.956), sex distribution (p=1.000), ASA status (p=1.000), or mean BMI (25.96±3.34 vs. 26.27±3.21, p=0.828). Mean CCI was not significantly different between cases and controls (4.90±1.81 vs. 4.90±1.81, p=1.000) nor was mean surgical duration (367.25±189.80 vs. 373.90±201.65, p=0.847). There was no significant difference between the cases and controls in HLOS (median (IQR) 2.0 (6) and 5.0 (7.00), p=0.473). The death rate was not significantly different between the cases and controls (36.4% vs. 9.1%, p=0.311), noting that the cases only had one death compared to four deaths in the controls. We were unable to assess the effect of HPV/p16 for the case-control analysis due to a lack of HPV/p16 data in four (36%) of the control subjects.

## Discussion

General anesthesia is widely used for many oncologic procedures, and numerous studies suggest that there may be some long-term survival benefits when using propofol infusions as a component of general anesthesia [9-17]. However, the improvement in overall survival and cancer recurrence is not uniform across all cancer types [18-20]. This retrospective study evaluated the relationship between propofol infusions and survival in patients with HNSCC. Based on the results of our study, there does not appear to be a survival benefit for patients who receive propofol infusions for maintenance anesthesia.

Our findings are in line with Miao et al., who conducted a similar study looking at survival in patients with different types of oral cancer who received either TIVA with propofol or inhaled anesthesia with sevoflurane [26]. In their study, they found that there was no significant difference in overall survival or cancer recurrence between groups in both primary and subgroup analyses. Our study largely corroborates these findings with several key distinctions. In their study, cohorts consisted of patients receiving exclusively propofol or exclusively inhaled anesthetics, whereas our groups consisted of patients who received exclusively inhaled anesthetics or propofol infusions with or without inhaled anesthetics. Although this change did not lead to statistical significance, we feel that our groups are more representative of the typical anesthetic techniques used for these types of surgeries, which often utilize a combination of inhaled and intravenous anesthetic agents.

Perhaps the most important difference between our studies relates to the inclusion of HPVp16 status for patients. Miao et al. did not analyze HPV/p16 status in their study. This variable is important in HNSCC because it significantly affects survival. In one study, there was a 15-20% improvement in 10-year survival for patients with HPV-positive tumors compared to patients with tumors negative for HPV [27]. Similarly, our study also demonstrated an improved mortality rate for patients with HPV-positive tumors (8% vs. 27%), although propofol infusions did not appear to contribute to this improvement. Furthermore, the prevalence of HPV-positive HNSCC varies significantly by geography. The population in the study by Miao et al. consisted of patients at a major Chinese medical center, whereas our population was taken from an academic institution in the United States. According to one study, HPV is responsible for 23% of oropharyngeal SCCs, 1.4% of oral cavity SCCs, and 1.1% of laryngeal SCCs in China, all below worldwide rates [28]. In contrast, HPV is responsible for 51%, 4.3%, and 4.6% of oropharyngeal, oral cavity, and laryngeal SCCs in North America, respectively. These are all well above the worldwide rates of HPV-positive tumors for each anatomical location. Therefore, it is likely that the majority of patients in Miao et al. had HPV-negative tumors. Because of this, it is not clear from their study whether propofol infusions improve mortality in HPV-positive HNSCC. Based on our results, it does not appear that HPV/p16 status influences HNSCC’s response to propofol infusions.

Some other major differences between our studies relate to where our data was gathered. We utilized the NDI, a federal database that catalogs individuals’ dates of death in the United States, to ensure that death dates were accurate, as they were often unreported in our EMR. Because the study by Miao et al. did not include a similar step, it is possible that their survival rates are overestimated due to incomplete data, as ours would have been without collecting data from the NDI. Finally, our analysis was not propensity score matched due to the small sizes of our groups, whereas Miao et al. did perform propensity score matching to address any potential confounders due to baseline characteristics.

In general, previous studies looking at the effects of propofol on cancer recurrence have demonstrated large effects. For example, in a study published by Wu et al., they found propofol improved survival in patients with colorectal cancer with hazard ratios as low as 0.22 in patients with lower TNM stages and 0.42 with higher TNM stages [9]. Similarly, Lai et al. found propofol improved survival for patients undergoing radical prostatectomy with a hazard rate of 0.11 [15]. These two studies illustrate how large the effects of propofol may be in other cancer types. Therefore, it is reasonable to expect that propofol infusions may have a similar effect size in HNSCC. Despite this, even a large effect would be difficult to detect with our sample size. We feel that our study is underpowered and unable to detect large differences in effect, such as those demonstrated in prior studies.

There are multiple limitations to our study. As mentioned, our sample size was small, and it may not have been able to detect even large effects. In addition, it is possible that the use of sevoflurane in both groups had a confounding effect on our data, as sevoflurane has been shown to both inhibit tumor progression in vitro [29] and promote the expression of pro-oncogenic tumor markers in vivo [30]. Despite these limitations, we believe this study provides insight into how HNSCC responds to propofol infusions. Further work still needs to be done in this area to fully characterize which cancers, including HNSCC, benefit from propofol infusions.

## Conclusions

HNSCC carries a high degree of morbidity and mortality, and surgical resection is one of the mainstays of treatment. In addition, HPV status plays a major role in the prognosis. Previous studies suggest that propofol infusions may provide a survival benefit for patients undergoing surgery for gastrointestinal tumors and various other types of cancer. The results of our study suggest that propofol infusions during surgery do not improve survival in patients with HNSCC undergoing surgical resection, regardless of tumor HPV status. Further research needs to be done in this area to better characterize the effects of propofol infusions on survival.
